# Club cell protein (CC)16 as potential lung injury marker in a porcine 72 h polytrauma model

**DOI:** 10.1007/s00068-022-01997-w

**Published:** 2022-05-21

**Authors:** Johannes Greven, Jan Tilmann Vollrath, Felix Bläsius, Zhizhen He, Eftychios Bolierakis, Klemens Horst, Philipp Störmann, Aleksander J. Nowak, Marija Simic, Ingo Marzi, Frank Hildebrand, Borna Relja

**Affiliations:** 1 Department of Trauma and Reconstructive Surgery, RWTH Aachen University, Aachen, Germany; 2 Department of Trauma, Hand and Reconstructive Surgery, Goethe University, Frankfurt, Germany; 3 Experimental Radiology, Department of Radiology and Nuclear Medicine, Otto-von-Guericke University, 39120 Magdeburg, Germany

**Keywords:** Uteroglobin, Inflammation, Biomarker, Tight junctions, Lung failure

## Abstract

**Background:**

Polytrauma and respiratory tract damage after thoracic trauma cause about 25% of mortality among severely injured patients. Thoracic trauma can lead to the development of severe lung complications such as acute respiratory distress syndrome, and is, therefore, of great interest for monitoring in intensive care units (ICU). In recent years, club cell protein (CC)16 with its antioxidant properties has proven to be a potential outcome-related marker. In this study, we evaluated whether CC16 constitutes as a marker of lung damage in a porcine polytrauma model.

**Methods:**

In a 72 h ICU polytrauma pig model (thoracic trauma, tibial fracture, hemorrhagic shock, liver laceration), blood plasma samples (0, 3, 9, 24, 48, 72 h), BAL samples (72 h) and lung tissue (72 h) were collected. The trauma group (PT) was compared to a sham group. CC16 as a possible biomarker for lung injury in this model, and IL-8 concentrations as known indicator for ongoing inflammation during trauma were determined by ELISA. Histological analysis of ZO-1 and determination of total protein content were used to show barrier disruption and edema formation in lung tissue from the trauma group.

**Results:**

Systemic CC16 levels were significantly increased early after polytrauma compared vs. sham. After 72 h, CC16 concentration was significantly increased in lung tissue as well as in BAL in PT vs. sham. Similarly, IL-8 and total protein content in BAL were significantly increased in PT vs. sham. Evaluation of ZO-1 staining showed significantly lower signal intensity for polytrauma.

**Conclusion:**

The data confirm for the first time in a larger animal polytrauma model that lung damage was indicated by systemic and/or local CC16 response. Thus, early plasma and late BAL CC16 levels might be suitable to be used as markers of lung injury in this polytrauma model.

## Introduction

Despite achievements to reduce injury-related mortality and morbidity in the last decades, the number of polytraumatized patients admitted to hospitals remains high with approximately 4.8 million human deaths worldwide caused by traumatic injuries every year [1]. In polytraumatized patients blunt thoracic trauma is common, and around 20–25% of deaths in severely injured patients treated in emergency departments are caused by chest injuries [2]. Furthermore, thoracic trauma has been shown to be associated with increased posttraumatic inflammation compared to polytrauma patients who did not undergo thoracic trauma, increased rates of pneumonia and Acute Respiratory Distress Syndrome (ARDS) and lower survival rates of patients [3–5]. It is of utmost importance for the medical community to detect changes in the lungs as early as possible to prevent acute lung injury (ALI).

Club cell protein (CC)16 is a 15.8 kDa homodimeric protein and the major protein secreted in airways by the non-ciliated bronchiolar Club cells [6, 7]. It occurs in very high concentrations in the epithelial lining where it appears to play an antioxidant and anti-inflammatory role [7]. Its role as a potential biomarker to detect pulmonary damage in polytraumatized patients or to identify polytraumatized patients at risk for secondary pulmonary complications has been extensively studied during the last decades. Wutzler et al. reported that in patients who have sustained multiple injuries, the early elevation of CC16 serum levels can be used to accurately identify patients with versus those without lung injury [8]. Furthermore, serum CC16-levels have been shown to correlate with the volume of lung contusion in polytraumatized patients [8], and were proposed to serve as a biomarker to monitor the progression of pulmonary contusion [9]. Negrin et al. investigated CC16 levels in polytraumatized patients with severe thoracic injury and reported that CC16 levels exceeding 30.51 ng/ml on day two after trauma may allow a firmer diagnosis for the development of pneumonia [10]. Another study reported a second peak in CC16 levels in patients developing pneumonia after polytrauma with severe thoracic trauma [11]. Xu et al. investigated ex vivo in vitro association of CC16 and polymorphonuclear leukocytes (PMNL) in polytraumatized patients with or without pneumonia and observed significant effects of CC16 on PMNL in patients with pneumonia [12]. The authors hypothesized that CC16 might reduce the migratory capacity of PMNL, and thus, modulate their function in patients with respiratory complications after trauma [12]. Several factors affecting circulating CC16 have been identified as, e.g. on the one hand chronic exposure to tobacco smoke has been reported to cause a decrease in CC16 levels [13], while on the other hand acute exposure of firefighters to fire smoke has been reported to increase CC16 serum levels [14]. Following the hypothesis that CC16 might indicate lung epithelial injury during polytrauma in a large animal model with high translational aspects, we investigated the biomarker character of CC16 in a standardized porcine trauma model consisting of lung contusion, liver laceration, tibial fracture, and hemorrhagic shock followed by fluid resuscitation and fracture fixation with external fixator.

## Materials and methods

### Animal care

All experiments were performed in accordance with the German legislation governing animal studies, following the “Principles of Laboratory Animal Care” [15]. Official permission was granted by the North Rhine-Westphalia State Office for Nature, the Environment and Consumer Protection (Landesamt für Natur-, Umwelt- and Verbraucherschutz Nordrhein-Westfalen, Recklinghausen, Germany, AZ 81.02.04.2018.A113). Male German landrace pigs were housed with a 12 h day/night rhythm at about 22 °C and allowed to acclimatize to their surroundings for a minimum of 7 days before the surgery. This experiment was part of a larger study of pigs that sustained polytrauma (PT group) and non-injured controls (sham group) (German Landrace, *Sus scrofa*). The pigs had a 35 ± 5 kg body weight (BW). For the current study, eight pigs from each group (sham and PT group) were included in the project. All animals underwent clinical examination and vaccination (21 days before experiments; circovirus and mycoplasma) by a veterinarian before the experiments started to exclude pre-infections. This study presents partial results obtained from a large animal porcine polytrauma model [16].

### General instrumentation and anesthesia

The experimental setup was established at the Department of Trauma Surgery, RWTH, Aachen in 2016 and can be found elsewhere [17]. In brief, anesthetized (propofol 2%/2 mg/kg BW/h i.v.) and analgized (fentanyl 40–90 μg/kg BW/h i.v.; additional midazolam 0.1–0.25 mg/kg BW/h to prevent shivering) male pigs were intubated, ventilated, and continuously monitored (electrocardiographic recording/pulse oximetry). All pigs were further monitored by arterial pulse contour cardiac output (PiCCO, Pulsion Medical Systems, Germany), and catheters were placed into the right femoral artery, the left femoral vein [two-lumen hemodialysis catheter (Arrow International, Germany)], the right jugular vein [central venous catheter (Arrow International, Germany)], and the bladder (suprapubic catheter). Instrumentation was applied under sterile conditions.

### Induction of trauma

For the induction of blunt thoracic trauma, a pair of steel and lead panels (each 5 × 10 cm in the surface, 0.8 and 1.0 cm thickness, respectively) was applied to the left dorsal lower chest. Blunt lung contusion was induced by a bolt gun machine (Blitz-Kerner, turbocut JOBB GmbH, Germany). Cattle-killing cartridges (green, 9 × 17; DynamitNobel AG, Troisdorf, Germany) were used. The bolt shot onto this panel simulated blunt lung contusion as previously described [17, 18]. O_2_ was defined at 21% during the trauma period, hence simulating the ambient air. An additional laparotomy was done to approach the liver to mirror a penetration injury. The midlobe of the liver was cut crosswise (3 cm) to half the liver thickness in depth, and uncontrolled bleeding was allowed for 30 s. After that, liver packing and suturing of the laparotomy was performed. Furthermore, a tibia fracture was carried out using a cattle gun. Pressure-controlled and volume-limited hemorrhagic shock was induced by withdrawing blood until a mean arterial pressure (MAP) of 40 ± 5 mm Hg was reached. Here, a maximum of 45% of total blood volume was drawn from the left femoral vein. The shed blood was kept in blood bags for reinfusion purposes. Hemorrhagic shock was maintained for 90 min. After this period, the animals were resuscitated in accordance with established trauma guidelines (ATLS^®^, AWMF-S3 guideline on Treatment of Patients with Severe and Multiple Injuries^®^), and the tibia fracture was fixed by external fixation [19, 20].

### Resuscitation phase

In addition to pre-warmed crystalloids (SterofundinISO and pediatric electrolyte solution 2–4 ml/kg BW/h), the animals received their previously withdrawn blood to restore hemostasis. The animals were mechanically ventilated and monitored on a special intensive care unit (ICU) for 72 h post-injury according to well-established ICU treatment guidelines (AWMF-S3 guideline on Treatment of Patients with Severe and Multiple Injuries^®^). The hemodynamics for this model have been described before [17]. After 72 h animals were euthanized using potassium chloride followed by harvest of the organs.

### Sham animals

Sham animals were not subjected to injury, laparotomy or hemorrhage but were identically instrumented and received the same anesthetic and intensive care procedures as the trauma animals.

### Blood processing and analysis

Blood samples were obtained immediately after implementation of the central venous catheter (0 h) and 3 h, 9 h, 24 h, 48 h and 72 h after polytrauma in prechilled ethylenediaminetetraacetic acid tubes (EDTA, S-Monovette^®^, Sarstedt, Nümbrecht, Germany) and kept on ice. Blood was centrifuged at 2000×*g* for 15 min at 4 °C and supernatant was stored at −80 °C until further analysis.

### CC16 and IL-8 in the bronchoalveolar lavage fluid

Bronchoalveolar lavage (BAL) was performed after collection of the right lung with 100 mL PBS using a perfusor syringe and a perfusor line. The obtained BAL was given into EDTA containing tubes (Sarstedt, Germany) stored oat 4 °C and subsequently centrifuged at 2200×*g* to measure the CC16 and IL-8 concentration in the remaining supernatant by ELISA. Samples were stored on −80 °C until measurement.

### CC16 levels in lung tissue

After lung perfusion with PBS via the pulmonary artery three pieces of the right lobe of the lung were immediately snap frozen using liquid nitrogen and then stored at −80 °C until sample analysis. For lung tissue sample analysis, lung tissues were crushed with the help of a mortar and pestle, previously cooled down with liquid nitrogen. The remaining tissue powder was incubated with RIPA lysis and extraction buffer (10 µl buffer/1 mg tissue; Thermo Fisher Scientific, Massachusetts, USA) for 15 min at 37 °C. The suspension was centrifuged at 2200×*g* for 15 min on 4 °C.

### ELISA measurements

ELISA were performed with BAL (CC16 and IL-8), lung tissue (CC16) and plasma samples (CC16) in accordance to the manufacturer´s manuals (MyBioSource, MBS741482, San Diego, USA and R&D Systems, P8000, Minneapolis, USA). To determine CC16 and/or IL-8 concentrations in BAL and plasma samples, those were spun down for 15 min at 2200×*g* at 4 °C and subsequently the supernatant was used for testing. Lung tissue samples were prepared as described above.

### Total protein determination in BAL

Protein concentrations were determined in BAL using the Pierce^®^ BCA Protein Assay Kit (Thermo Scientific) following the manufacture’s manual.

### ZO-1 immune histology of lung tissue

The left lobe of the lung was flushed with 4% formalin for overnight fixation and kept in 70% ethanol until paraffin embedding and sectioning. Paraffin-embedded lung samples (left lobe) were sectioned (3 µm), deparaffinized, rehydrated and stained with anti-ZO-1 antibody (Affinity Biosciences, USA). Following deparaffinization, antigen retrieval was done under steam atmosphere using R-Universal epitope recovery buffer (Aptum, Kassel, Germany) for 1 h (Retriever 2010, Prestige Medical). The endogenous tissue peroxidase activity was blocked with hydrogen peroxide according to the manufacturer’s instructions (Peroxidase UltraVision Block, Dako). After washing with water and PBS, Anti-ZO-1 antibody (1:100, Cat.#: AF5145) was applied as a primary antibody. After the incubation for one hour at room temperature, and a subsequent washing procedure, a secondary antirabbit horseradish peroxidase-linked antibody (Nichirei Biosciences Inc.) was applied to detect specific binding. As substrate, 3-amino-9-ethylcarbazole (AEC, DCS Innovative Diagnostik-Systeme, Hamburg) was applied. Then, the sections were counterstained with hematoxylin. The relative staining intensity of the AEC substrate per slide was evaluated using the ImageJ software in a blinded manner by an independent examiner.

### Statistical analysis

GraphPad Prism 6.0 software (GraphPad Software Inc. San Diego, CA, USA) and BM SPSS Statistics 27 (IBM, New York, USA) were used to perform the statistical analyses. The data distribution was tested by the D'Agostino-Pearson test to test for normal distribution. For repeated measures, the non-parametric Friedman`s test was applied. Dunn–Bonferroni post hoc test for multiple comparisons was used. The comparison between polytrauma and sham groups was performed using the unpaired *T* test. Data are given as mean ± standard error of the mean (SEM). A *p* value below 0.05 was considered statistically significant.

## Results

### Systemic CC16 course after polytrauma

**Fig. 1:**
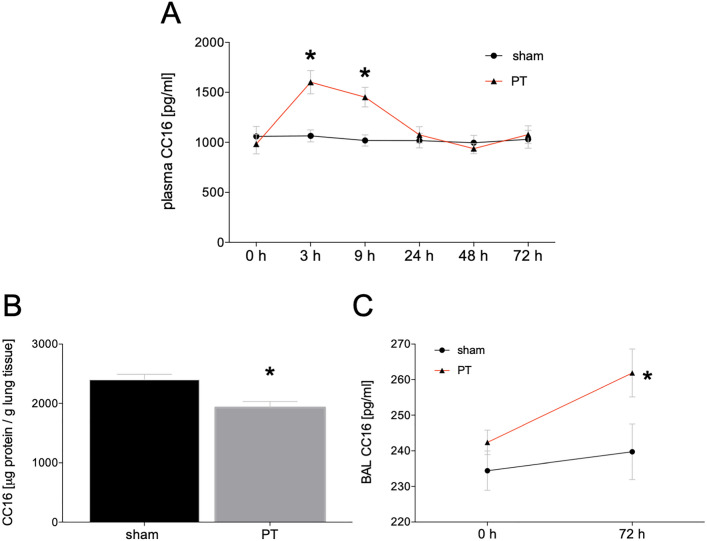
Time course of mean concentrations of club cell protein (CC)16 in plasma samples. Blood samples were obtained before (0 h) trauma, and 3 h, 9 h, 24 h, 48 h, and 72 h after (**A**). Summary of mean concentrations of CC16 in lung tissue (**B**) and bronchoalveolar lavage (BAL) fluid (**C**). BAL fluid was collected before (0 h) trauma and 72 h after polytrauma. Lung tissue was collected 72 h after polytrauma. Sham is the control group (*n* = 8), polytrauma (PT) animals underwent PT (*n *= 8). The results are represented as the mean ± SEM, *p < 0.05 vs. sham

The time course of CC16 concentrations in the comparison of PT and sham animals has shown a clear difference between the two groups by significantly increased values in the polytrauma group at 3 h and 9 h after trauma compared to sham (3 h: 1601.1 ± 116.0 pg/g vs. 1065.71 ± 99.93 pg/g; 9 h: 1452.13 ± 98.67 pg/g vs. 1018.68 ± 56.36 pg/g; *p* < 0.05, Fig. 1A).

### CC16 expression in lung tissue and BAL

The comparison of the CC16 concentration in lung homogenates has shown significantly higher CC16 levels in the lung tissue of sham animals compared to PT (2393.32 ± 219.14 µg/g vs. 1929.55 ± 253.36 µg/g; *p* < 0.05, Fig. 1B). CC16 levels in the BAL from polytraumatized animals were significantly enhanced compared to sham animals at 72 h after polytrauma (*p* < 0.05, Fig. 1C).

### Local inflammatory changes in BAL

**Fig. 2:**
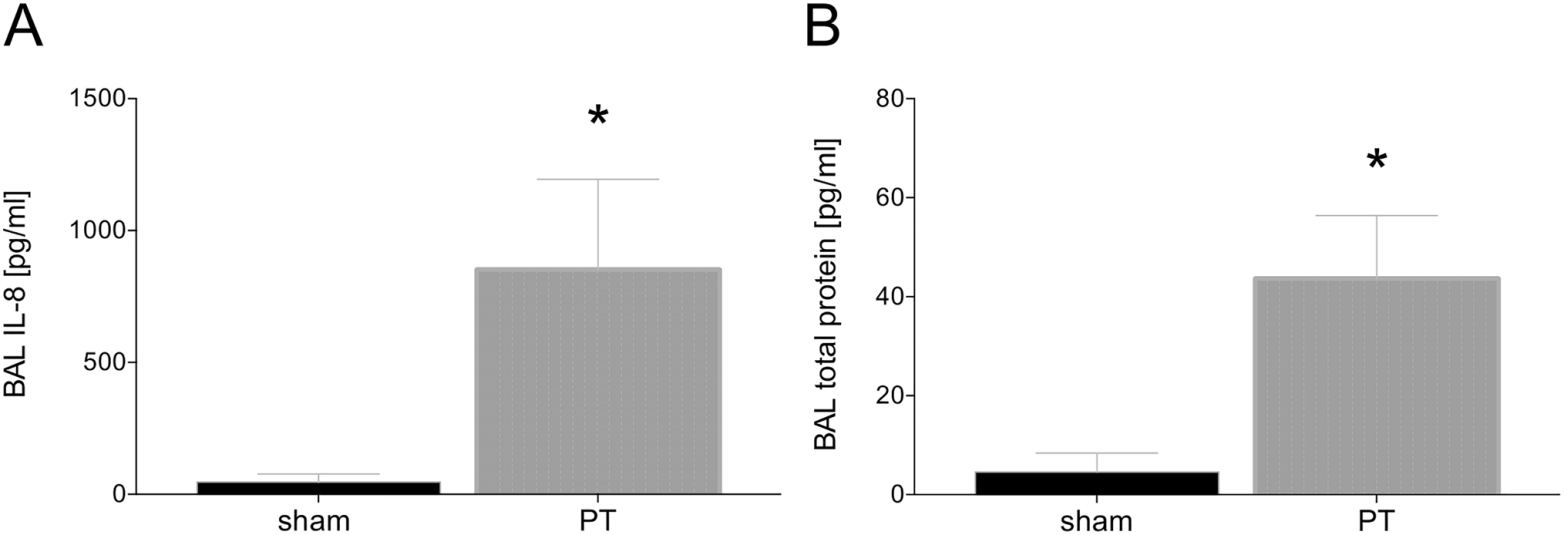
Summary of mean concentrations of interleukin (IL)-8 (**A**) and total protein concentration (**B**) in the bronchoalveolar lavage (BAL) fluid. BAL fluid was collected at 72 h after polytrauma. Sham is the control group (*n* = 8), polytrauma (PT) animals underwent PT (*n *= 8). The results are represented as the mean ± SEM, *p < 0.05 vs. sham

The measurement of IL-8 and total protein concentrations in BAL of sham and PT animals revealed that IL-8 and total protein content were significantly higher in the PT group compared to sham at 72 h after polytrauma (*p* < 0.05, Fig. 2A, B).

### Examination of lung injury and epithelial barrier breakdown after polytrauma

**Fig. 3:**
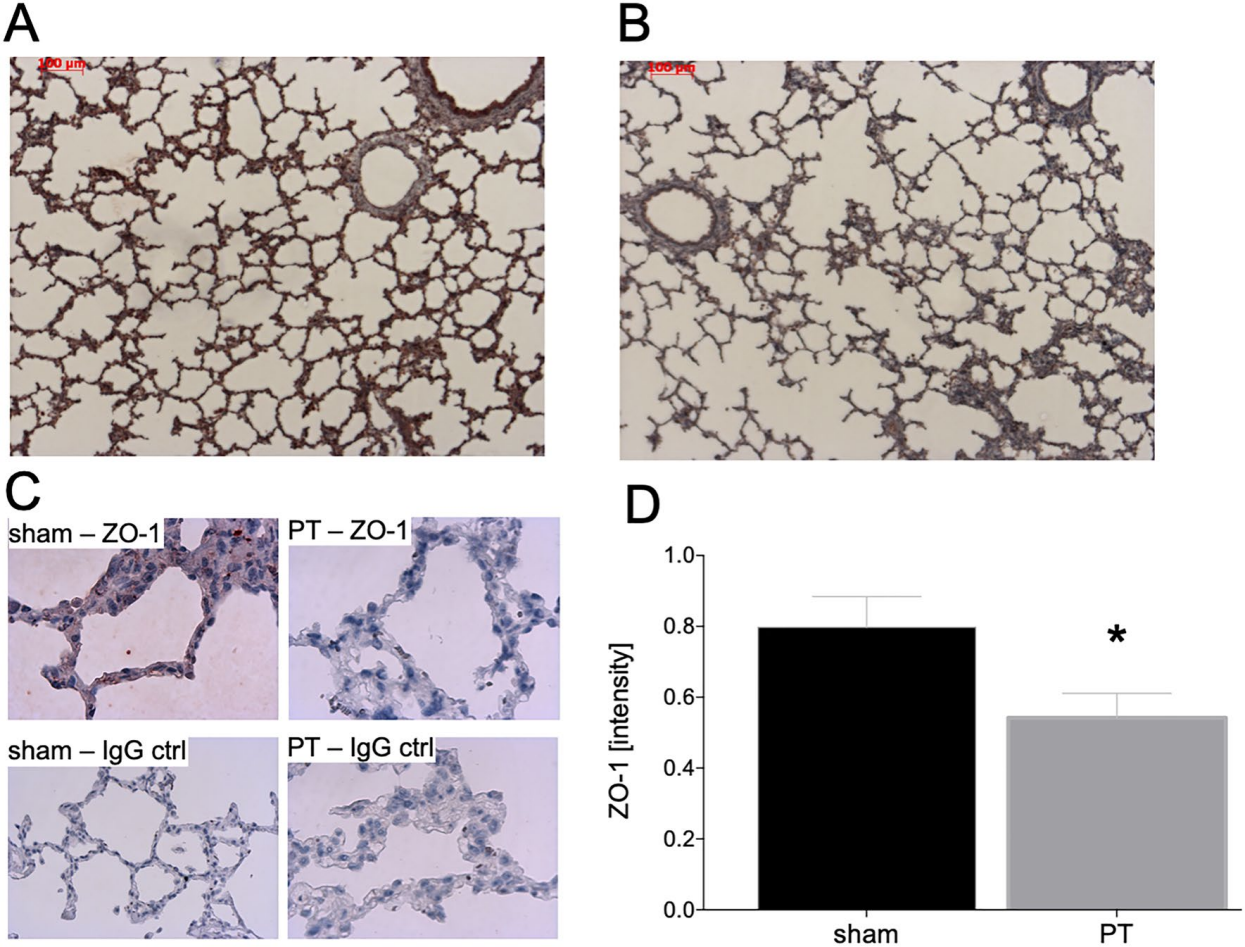
Histological evaluation of zonula occludens (ZO)-1 staining 72 h after polytrauma. Sham operated animals underwent the same surgical procedures but polytrauma (PT) was not carried out. Representative ZO-1-stained lung sections from sham (**A**) and PT (**B**) groups are shown. Control staining with the IgG control antibody is shown in all groups (**C**). The protein expression intensity was quantified (**D**). Sham is the control group (*n* = 8), polytrauma (PT) animals underwent PT (*n* = 8). The results are represented as the mean ± SEM, bar is 100 µm, *p < 0.05 vs. sham

The evaluation of lung injury and loss of tight junctions by immune histological staining with a ZO-1 specific antibody has shown that the sham group had a significantly higher ZO-1 protein expression intensity compared to the PT group (*p* < 0.05, Fig. 3D).

## Discussion

Elevated systemic CC16 levels in conditions of acute as well as chronic lung damage have been shown before [8, 14, 21]. Nevertheless, there are still scientific debates about which markers are reliable, especially in multiple injuries, to assessing lung injury. Many of these markers were not specific enough or were subject to major changes in their expression levels regarding the overall damage, a disease state/pre-existing conditions, or the etiology and have been influenced by the time of measurement [22–26].

Our results clearly indicated that CC16 has been significantly changed both locally in the lung tissue as well as systemically after polytrauma with thoracic injury. Interestingly, the systemic levels of CC16 increased very early after polytrauma. These findings are in direct agreement with data from Wutzler et al. and Lin et al. [11, 27]. In these studies, an early increase within the first hours after trauma was detected reaching baseline levels within 24 h again. In addition, Wutzler et al. have shown a second CC16 increase in sera obtained from traumatized patients who developed pulmonary complications [11]. The time course of the CC16 increase and the renewed decrease to baseline level within 24 h was reproduced in the underlying polytrauma model. Due to multiple sampling time points of plasma before and directly after trauma induction, resulting in a higher temporal resolution, it has been shown that the concentrations’ peak was around 3 h after trauma with baseline values being reached again after 24 h. Whether this was dependent on the trauma severity, or the intensity of lung damage remains to be investigated in further studies. The secondary increase of CC16 serum levels in clinically later occurring respiratory problems has been described by Wutzler et al., and was not reproduced in our 72 h model, but is also might not be expected within this short ventilation period in our model [11]. Prolonged observational time points are necessary to validate the clinically observed relevance of CC16 as a potential indicator for the secondary lung complications after trauma.

Another interesting effect regarding the different concentrations of CC16 over time in the different compartments might have occurred due to a short-term high, but then decreasing and persisting release of CC16. While CC16 increased very quickly systemically and decreased after a short peak, CC16 was only significantly increased in the BAL at 72 h after polytrauma. Furthermore, CC16 was present in lower concentrations in the lung of polytraumatized animals after 72 h compared to sham animals. These differences may have occurred due to an initial storage dependent release, which was possibly followed by a synthesis and expression dependent release.

Lung damage or respiratory complications are often driven by inflammatory changes involving the proinflammatory cytokine IL-8. The above described significantly increased values of IL-8 in the BAL obtained from polytraumatized animals have been associated with the loss of pulmonary barrier function, and a CC16 increase found in polytraumatized animals. In parts this has been confirmed by Reynolds et al. who demonstrated in their mouse model over-expressing transgenic human IL-8 that the prolonged presence of high IL-8 concentrations, as observed in trauma, exerted a damaging effect to the lung [28]. The authors demonstrated modifications in the context of long-term structural damage, with damaged and leaky epithelial tight junctions. Yang et al. showed that this might have been based on surfactant A and B expression depending on IL-8 occurrence [29]. In addition, Lin et al. have demonstrated that CC16 levels at ICU admission directly correlated with the severity of ARDS, but they only provided limited information for prognostic purposes [27]. In the present study, lung injury has been confirmed by increased total protein content in the BAL, which indicated functional disruption of the lung barrier. This is in line with the findings of Herrero et al. who found a direct correlation between ZO-1, loss of barrier function and edema formation in their mouse model [30]. In our study, sham animals have shown significantly higher values of membrane-bound ZO-1, which represented a more intact and functional lung barrier here. Taken together, our findings indicate that CC16 may constitute as an early biomarker for the pulmonary barrier loss that is associated with increased lung injury in this large animal polytrauma model, thus suggesting that this model reflected the pathophysiological modulations that have been observed in the human situation of polytrauma. Furthermore, this study provides insights into changes of CC16 levels in lung tissue after polytrauma, which is not possible to be investigated in human clinical studies.

## Limitation

Since the measurements were made exclusively in comparison of a polytrauma to a sham group and no isolated thoracic trauma group was included, the influence of other organ injuries, cannot be completely excluded. The characteristics as a lung epithelial injury marker can only be drawn by comparison to other animal models. Therefore, to be able to make a definitive statement, further investigations would have to be carried out taking into account an isolated thoracic trauma group including different degrees of trauma and outcome-oriented longer time courses. Furthermore, further studies need to be undertaken to evaluate the informative value of CC16 and its expression patterns under pre-disease conditions. Nevertheless, our study indicates that CC16 might also be used as a marker for lung injury in the porcine model of polytrauma. The potentially lung-protective inhalation of CC16 would be a worthy further study to consider if this is a therapeutic target. The temporal limitations to 72 h of the monitoring phase do not allow conclusions to be drawn about unaffected outcome at this time, or the development of later occurring organ and/or multiple organ failure in this model. Further studies would need to pay more attention to important cell populations such as e.g. neutrophils and other immune-related cells.

## Conclusions

Our data demonstrated that CC16 may be used as a potential marker of lung injury in this porcine polytrauma model. Early increased systemic CC16 levels indicated lung damage after polytrauma, while the local CC16 increase in BAL may be used as a later marker even 3 days after polytrauma to indicate the lung injury which is associated with the barrier breakdown and inflammatory changes.

## Data Availability

The data are available upon a reasonable request from the corresponding author.
